# Effects of early mobilization on short-term blood pressure variability in acute intracerebral hemorrhage patients: A protocol for randomized controlled non-inferiority trial

**DOI:** 10.1097/MD.0000000000026128

**Published:** 2021-05-28

**Authors:** Hsiao-Ching Yen, Jiann-Shing Jeng, Chiung-Hua Cheng, Guan-Shuo Pan, Wen-Shiang Chen

**Affiliations:** a Division of Physical Therapy, Department of Physical Medicine and Rehabilitation; b Stroke Center & Department of Neurology; c Department of Physical Medicine and Rehabilitation, National Taiwan University Hospital, Taipei, Taiwan.

**Keywords:** blood pressure variability, early mobilization, intracerebral hemorrhage, neurointensive care unit, physical therapy

## Abstract

**Background:**

Early out-of-bed mobilization may improve acute post-intracerebral hemorrhage (ICH) outcomes, but hemodynamic instability may be a concern. Some recent studies have showed that an increase in mean systolic blood pressure (SBP) and high blood pressure variability (BPV), high standard deviation of SBP, may lead to negative ICH outcomes. Therefore, we investigated the impact of an early mobilization (EM) protocol on mean SBP and BPV during the acute phase.

**Methods:**

The study was an assessor-blinded, randomized controlled non-inferiority study. The participants were in An Early Mobilization for Acute Cerebral Hemorrhage trial and were randomly assigned to undergo EM or a standard early rehabilitation (SER) protocol within 24 to 72 hour after ICH onset at the stroke center. The EM and SER groups each had 30 patients. 24-measurement SBP were recorded on days 2 and 3 after onset, and SBP were recorded three times daily and during rehabilitation on days 4 through 7. The two groups’ mean SBP and BPV under three different time frames (days 2 and 3 during the acute phase, and days 4 through 7 during the late acute phase) were calculated and compared.

**Results:**

At baseline, the two groups’ results were similar, with the exception being that the mean time to first out-of-bed mobilization after symptom onset was 51.60 hours (SD 14.15) and 135.02 hours (SD 33.05) for the EM group and SER group, respectively (*P* < .001). There were no significant differences in mean SBP and BPV during the acute and late acute phase between the two groups for the three analyses (days 2, 3, and 4 through 7) (*P* > .05).

**Conclusions:**

It is safe to implement the EM protocol within 24 to 72 hour of onset for mild-moderate ICH patients during the acute phase.

## 1 Introduction

Early mobilization (EM) refers to out-of-bed mobilization within 72 hour of admittance to an intensive care unit, and it is important to restoring function based on neuroplasticity mechanisms after stroke.^[1,2]^ Out-of-bed mobilization enables patients to participate in upright and task-specific activities that increase verticalization stimulation, which stimulates the afferent sensory system and provides more motor experience enrichment that could improve functional recovery.^[3–7]^ However, patients with spontaneous intracerebral hemorrhage (ICH) are mostly mobilized later than patients with ischemic stroke during the acute phases^[8,9]^ due to impaired cerebral autoregulation after ICH. This may increase systolic blood pressure (SBP) and disrupt the blood-brain barrier, resulting in vasogenic perihematomal edema during the acute phase.^[10]^ Therefore, clinicians often struggle to conduct early out-of-bed mobilization programs due to the perceived lack of evidence, as well as the fear of hemodynamic instability and occurrence of re-bleeding events in a stroke center.

Recent findings not only suggest that having high SBP after acute ICH is significantly associated with hematoma expansion and unfavorable outcomes,^[11–13]^ but also that blood pressure variability (BPV) may independently influence hemorrhage growth, recurrence, edema occurrence, and outcomes at two to seven days.^[11,14,15]^ Cerebral blood flow is regulated during rapid SBP changes and BPV indicates as static autoregulation that occurs within minutes to hours in response to changes in SBP.^[16,17]^ Therefore, disrupted cerebral autoregulation mandates strict control of SBP after ICH through anti-hypertensive treatments during the acute phase.^[18]^ However, depending on its intensity and type, out-of-bed mobilization could result in rising noradrenaline and adrenaline plasma concentrations that increase SBP^[19]^ or affect BPV stability. There is rare evidence from randomized trials regarding the stability of mean SBP and BPV when implementing an EM protocol at two to seven days after ICH onset.

Therefore, we assessed the clinical significance of the mean SBP and BPV within seven days after onset in patients with ICH who participated in the An Early Mobilization for Acute Cerebral Hemorrhage (AEMACH) trial at a stroke center.^[20]^ The aim of this study was to examine whether an early out-of-bed mobilizaton protocol could be implemented in a stroke center without causing significant changes in mean SBP and inter-individual BPV during the acute phase.

## 2 Methods

### 2.1 Study design

This study was a prospective, assessor-blinded, and randomized controlled non-inferiority trial with 2 parallel groups and the collected data was subjected to a secondary analysis that used data from the AEMACH trial.^[20]^ The randomized controlled AEMACH trial was conducted to examine the usefulness of initiating an early mobilization protocol within 24–72 h after onset of spontaneous ICH at the stroke center of the largest medical center in Taiwan. The intervention time and daily session duration were similar to those of standard care and a standard early rehabilitation (SER) approach focused on restoring the motor domain of functional independence measures^[21,22]^ by three months after stroke. The study protocol was approved by the Institutional Review Board of the National Taiwan University Hospital (201706073RINB) and registered at ClinicalTrials.gov (trial registration ID: NCT03292211). All of the participants provided written informed consent before being subjected to randomization. All research procedures were conducted in accordance with the ethical standards of the Declaration of Helsinki.

### 2.2 Participants

The inclusion criteria in the study were as follows: having had a first primary ICH occurrence with unilateral hemiparesis/hemiplegia that was confirmed via computed tomography; not having failed the set physiological safety limits for mobilization within 72 hour of stroke onset (100 < SBP <160 mm Hg at rest, 40< resting heart rate <130 bpm, oxygen saturation >92% without supplementation, and no hydrocephalus or raised intracranial pressure (>20 mm Hg) before intervention); a National Institutes of Health Stroke Scale score of <20 at admission; complete pre-stroke independence in activities of daily living; and being aged 20 to 80 years. However, patients were excluded if they had ICH due to trauma, surgery, a hemorrhagic transformation from stroke, or an underlying mass; other medical conditions preventing mobilization; or a rapid early deterioration of symptoms within 24 h of stroke onset.

### 2.3 Intervention

The EM and SER groups underwent different rehabilitation protocols.^[20]^ The protocol applied to the EM group was functional and involved sitting unsupported on the edge of the bed by rolling, sitting up for a minimum of 15 minutes, and then standing with/without hand support for approximately 3 to 5 minutes, whenever possible.^[20]^ During their stay at the stroke center, the participants in the SER group focused on performing training activities in bed, which included joint range-of-motion, bridging, straight leg-raising, and stretching exercises; facilitation techniques; and rolling and sitting for bed training (sitting in bed). In the SER group, out-of-bed mobilization was started as soon as possible after intensive care unit discharge and was adjusted and implemented according to each participant's ability and progress. Both groups received care five days a week, with one 30-minutre session being conducted daily until discharge from acute stroke care. The mean time to mobilization after symptom onset (defined as the time elapsed from admission to out-of-bed unsupported sitting) was 51.60 hours for the EM group and 135.02 hours for the SER group.^[20]^

### 2.4 Data collection

The SBP data were collected within seven days after ICH onset. On day 2 and day 3 after ICH, SBP was recorded hourly (with an automated electronic device worn around the nonparetic arm) at 23 time points by a nurse at the stroke center. Another BP reading was also taken during rehabilitation, after which an ER group participant would spend 5 minutes sitting at the edge of a bed under the supervision of a physical therapist, while an SER group participant would spend 5 minutes performing an in-bed activity. For the next four to seven days after ICH, SBP levels were recorded by a stroke center or general ward nurse three times daily (morning, afternoon, and evening) with an automated electronic device. SBP levels on days 4 through 7 after ICH onset were also recorded during rehabilitation, which was followed by 5 minutes of intervention for both groups every day. Data relating to the incidence of hypotension (SBP<100 mm Hg) and serious intervention-related adverse events during rehabilitation were recorded.

### 2.5 Outcome measurements

In addition to mean SBP, the other primary outcome in the study was BPV during the acute phase. Commonly used in related research, BPV is directly related to mean SBP, easy to measure, and significant for clinical practice.^[11,23]^ It is calculated using the standard deviation of SBP.

BPV and mean SBP were analyzed under the three different time frames as follows: day 2 after onset during the acute phase, day 3 after onset during the acute phase, and days 4 through 7 after onset during the late acute phase. Three different analyses were used to assess how the phase that the patients were in (acute or late acute phase) after ICH onset could potentially affect their outcomes. Therefore, the first and second analyses were performed for the acute phase, during which the SER and EM protocols were started at the stroke center. Mean SBP and BPV (standard deviation of the SBP) were calculated using 24 blood pressure measurements taken on days 2 and 3. The third analysis was performed for the late acute phase (days 4 through 7) during which the patients were discharged from the stroke center and transferred to a general ward. The mean SBP and BPV for the late acute phase were estimated using the SBP measurements obtained on days 4 through 7 (or until discharge).

### 2.6 Sample size calculation

Based on the results of the ASCOT-BPLA trial, an appropriate margin of non-inferiority for this trial is a decrease in the variability of systolic blood pressure from 10 to 8%, which should be regarded as clinically significant as it is associated with a reduction in stroke incidence.^[24]^ Therefore, for the calculations, a 10% non-inferiority margin, 90% power, and 2.5% one-sided level of significance were assumed. Accordingly, 99% of the participants in the SER group, and 90% of those in the EM group were assumed to have controlled BPV levels in the two parallel-sample designed trail. Each group must have approximately 30 participants in order to detect a non-inferior difference in BPV stability between the two groups.

### 2.7 Randomisation and procedures

Of the 89 patients who were screened, 60 were subsequently included and randomized at a ratio of 1:1 into the EM group and the SER group.^[20]^ These 60 participants were enrolled at the stroke center of the National Taiwan University Hospital ≤24 h after ICH onset for intensive antihypertensive treatment, which consisted mainly of intravenous nicardipine infusion based on Joint National Committee guideline recommendations.^[18]^ The 60 participants received a BP-lowering treatment with the primary aim of achieving an SBP of 130 to 140 mmHg within 1 hour of starting treatment and maintaining this SBP level for at least 7 days, which is recommended by the NICE guidelines released in 2019.^[25]^ The patients initially received intravenous agents but subsequently switched to oral agents due to drug availability. The objective was to maintain their SBP levels until they were transferred to the general ward.

### 2.8 Statistical analyses

The results were analyzed using the software SPSS (IBM SPSS Statistics 22, Chicago, IL). Descriptive statistics were expressed as mean (standard deviation) and median (interquartile range) values for continuous and ordinal variables, respectively, while number (n) and percentage (%) were used for categorical variables. Descriptive statistics pertaining to relevant patient characteristics were also compiled and reported. All continuous variables were first assessed for normality using the Shapiro–Wilk test. The independent t-test was used to compare the mean SBP between groups. The Mann–Whitney *U* test was used to compare the two groups’ BPV parameters with non-normal distribution. A two-sided *P* <.05 was considered to be significant.

## 3 Results

**Figure 1:**
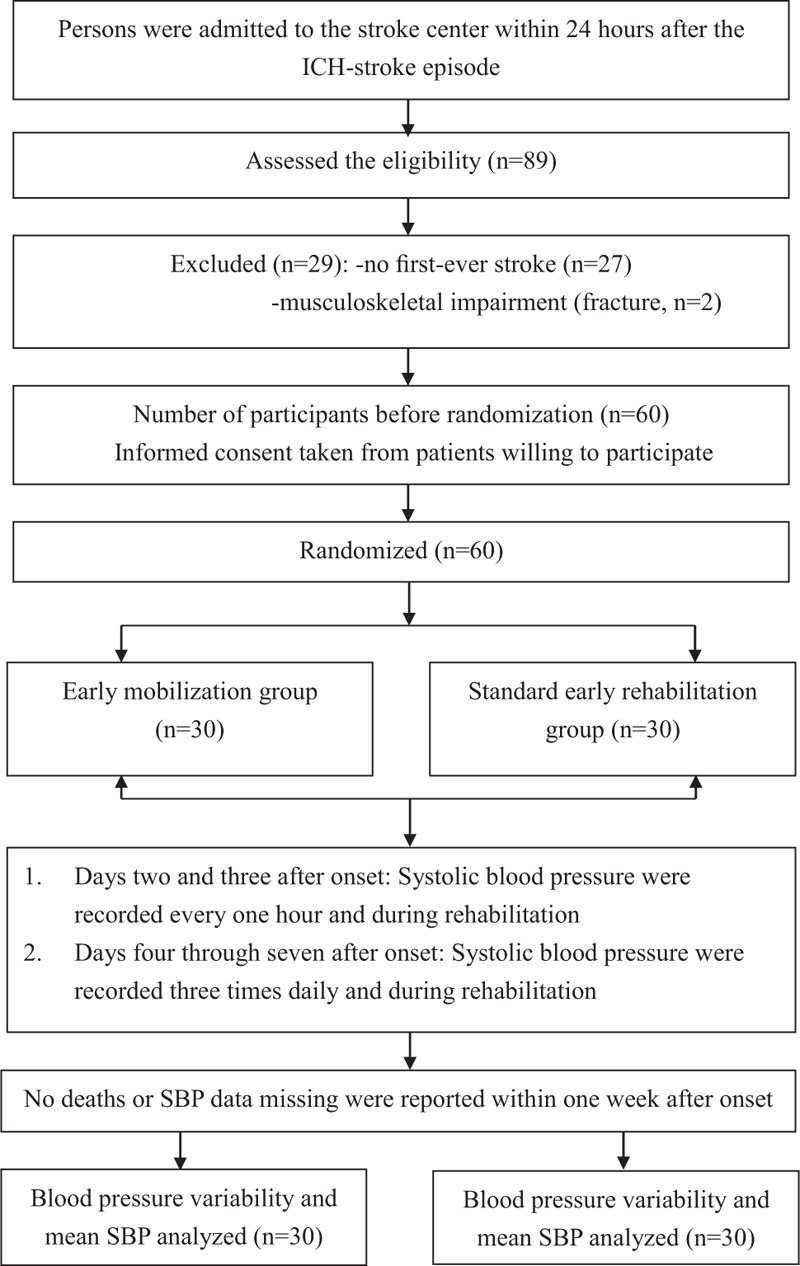
Flow diagram.

A flow diagram is presented in Figure 1. The 60 AEMACH trial participants were randomized into the EM group and the SER group, with each having 30 participants. There were no dropouts or missing BP measurements during the seven days after ICH onset, thus, all 60 patients were included in this analysis. Table 1 shows the participants’ attributes as recorded at the time of enrollment, which include demographic and clinical attributes and outcome measures. At baseline, the two groups were similar in terms of age, height, weight, sex, hematoma volume, stroke site, lesion location, initial National Institutes of Health Stroke Scale scores, and ICH score^[26]^ at admission, with the exception being the mean time to first mobilization after onset. Although the percentage of participants with dyslipidemia (a baseline stroke risk factor) in the SER group and the EM group participants was 36.7% and 20%, respectively, no significant difference was observed.

**Table 1:** Participant characteristics.

Group	SER (n = 30)	EM (n = 30)	*P*-value
Age (yr), mean±SD	59.33±13.13	58.77 ± 11.68	*P* = .860^a^
Height (cm), mean±SD	165.00 ± 9.34	164.73 ± 8.31	*P* = .907^a^
Weight (kg), mean±SD	68.67 ± 17.45	71.01 ± 17.41	*P* = .606^a^
Sex (female/male)	08:22	09:21	*P* = .774^b^
Lesion site (right/left)	11:19	14:15	*P* = .400^b^
The ICH score (0/1)	26:04	24:06	*P* = .488^b^
ICH location (supratentorial/infratentorial)	27:03:00	27:03:00	*P* = 1.000^b^
ICH volume (ml), mean±SD	10.03 ± 6.33	6.65 ± 7.95	*P* = .073^a^
Time to first mobilization (hours), mean±SD	135.02 ± 33.05	51.60 ± 14.15	*P* <.001^∗^^a^
NIHSS scores at admission, mean±SD	8.17 ± 4.62	6.33 ± 3.75	*P* = .097^a^
Hypertension, n (%)	26 (86.7%)	24 (80%)	*P* = .488^b^
Diabetes mellitus, n (%)	10 (33.3%)	6 (20%)	*P* = .243^b^
Dyslipidemia, n (%)	11 (36.7%)	6 (20%)	*P* = .152^b^
Current smoking, n (%)	2 (6.7%)	6 (20%)	*P* = .129^b^
Current heavy drinking, n (%)	1 (3.3%)	5 (16.7%)	*P* = .009^b^

At the early acute phase, the BPV on day 2 was 0.58 mm Hg higher in the SER group and 0.73 mm Hg higher in the EM group on day 3, but these differences were not significant (Table 2). The mean SBP analysis for both days 2 and 3 revealed similar results for the two groups (Table 2). At the late acute phase (i.e., days 4 through 7 after onset), there were also no significant differences in BPV and mean SBP between the two groups, hence establishing non-inferiority.

**Table 2:** The mean systolic blood pressure and blood pressure variability at days 2 and 3, and 4 to 7 between two groups.

	Mean systolic blood pressure, mm Hgmean (standard deviation)			Blood pressure variability, mm Hgmedian (interquartile range)		
Time	SER	EM	*P*-value^a^	SER	EM	*P*-value^b^
	N = 30	N = 30	(SER vs. EM)	N=30	N = 30	(SER vs. EM)
Day 2	137.53 (10.91)	137.38 (10.00)	.958	11.21 (9.15, 15.00)	10.63 (8.60, 13.00)	.469
Day 3	137.38 (10.04)	135.93 (14.52)	.338	10.64 (8.56, 13.38)	11.37 (10.01, 13.88)	.322
Days 4–7	132.22 (10.20)	135.99 (11.25)	.179	11.08 (8.23, 13.08)	10.98 (9.96, 12.94)	.626

## 4 Discussion

The minimal differences between the two groups, in regard to increases in BPV and mean SBP, indicate that the implementation of the early mobilization protocol within 24 to 72 hour after ICH onset is safe during the acute phase at a stroke center.

Impaired cerebral autoregulation is associated with unfavorable outcomes following ICH^[17]^ so mobilizing critically ill patients with hemorrhagic stroke can be difficult due to patient safety concerns relating to their hemodynamic status.^[27]^ However, in clinical practice, it is difficult to directly assess the status of cerebral autoregulation in acute ICH patients through continuous monitoring using transcranial Doppler sonography. Therefore, no studies have evaluated the impact of early mobilization intervention on blood pressure fluctuations or cerebral autoregulation during the acute phase. A recent study by Manning et al. demonstrated that SBP variability confers a poor outcome in acute ICH and concluded that SBP peaks are likely to have harmful effects.^[11]^ Since the early mobilization protocol in our study involved sitting and standing positions, for which body posture is a significant factor in autonomic activity,^[28]^ measuring BPV and mean SBP may be a clinically feasible means of obtaining insights into the impact of early out-of-bed mobilization on blood pressure fluctuations. Similar values in BPV and mean SBP outcomes were observed between the two groups in our study, which indicates that the SBP stability achieved by the early mobilization protocol was not inferior to that achieved by the in-bed early rehabilitation intervention.

In addition, early studies that utilized radiotracers have suggested that static cerebral autoregulation was preserved in intraparenchymal hemorrhage.^[29]^ Although both groups in our study received antihypertensive therapy in the acute period after ICH onset, the analysis of BPV and mean SBP in the acute period could have been potentially confounded by medication effects and other management decisions,^[30]^ and the implementation of the early mobilization protocol did not lead to inferior BPV and mean SBP inferior. Furthermore, since cerebral autoregulation impairment appears related to clinical severity (mean National Institutes of Health Stroke Scale score=31) and hematoma volume,^[31]^ this finding from our study may be also related to the presence of participants with mild-moderate clinical severity and low hematoma volumes. In addition, mobilization could adversely affect intracranial pressure in the early post-ICH period, but the inclusion criteria in the study had excluded patients with hydrocephalus or raised intracranial pressure. The exclusion of patients with raised intracranial pressure may partly explain why mean SBP and BPV were not significantly different between the two groups. Accordingly, the results cannot be generalized to patients with more severe ICH or those with high intracranial pressure. Moreover, cerebral autoregulation impairment may be more problematic in stroke patients with comorbid diabetes, as observed in type II diabetes studies.^[32]^ In the AEMACH trial, the rate of comorbid diabetes was relatively low; 33.3% of the SER group participants and 20% of the EM group participants had diabetes. In other words, it may be necessary to establish appropriate screening criteria for safe early mobilization interventions.

One important clinical implication of this study is that the early mobilization protocol's implementation after acute ICH onset may be feasible during the early rehabilitation process. One of the study's strengths was that it controlled for the type and frequency of early mobilization activity, as well as the frequency and duration of mobilization. However, the study has several limitations. First, data on cerebral blood flow during mobilization or dynamic cerebral autoregulation, which were derived from transient and sudden rapid changes in SBP, were not assessed. Second, the type of antihypertensive agent that is used can also affect BPV. Visit-to-visit variability in SBP was reportedly greater with β-blockers than with calcium-channel blockers (nicardipine/perdipine) in treated hypertensive patients.^[33]^ Third, patients with mild-to-moderate ICH stroke should be included to determine the applicability of the results to the overall population of ICH patients. Fourth, our study was based on a small sample from a single hospital. Further studies are needed to investigate intracerebral or cerebral perfusion pressure variations during early mobilization in patients with acute ICH, or moderate-severe ICH patients could also be included.

## 5 Conclusion

Our study showed that it is safe to implement the early mobilization protocol within 24 to 72 hours after ICH onset. Therefore, we recommend that stroke centers implement the early out-of-bed early mobilization for mild-moderate ICH patients without raised intracranial pressure.

## Acknowledgments

The authors wish to thank the staff at the stroke center of National Taiwan University Hospital for supporting this study.

## Author contributions

Hsiao-Ching Yen performed the literature search, data extraction and drafted of the manuscript; Hsiao-Ching Yen, Chiung-Hua Cheng and Guan-Shuo Pan collected the data; Jiann-Shing Jeng and Wen-Shiang Chen gave lots of suggestions; Hsiao-Ching Yen and Jiann-Shing Jeng designed the study. All authors approved the final manuscript.

**Conceptualization:** Hsiao-Ching Yen, Jiann-Shing Jeng, Wen-Shiang Chen.

**Data curation:** Hsiao-Ching Yen, Chiung-Hua Cheng, Guan-Shuo Pan.

**Formal analysis:** Hsiao-Ching Yen, Guan-Shuo Pan.

**Investigation:** Hsiao-Ching Yen, Jiann-Shing Jeng, Chiung-Hua Cheng, Guan-Shuo Pan.

**Methodology:** Hsiao-Ching Yen, Chiung-Hua Cheng.

**Project administration:** Hsiao-Ching Yen, Jiann-Shing Jeng, Wen-Shiang Chen.

**Resources:** Jiann-Shing Jeng, Chiung-Hua Cheng.

**Supervision:** Jiann-Shing Jeng, Chiung-Hua Cheng, Wen-Shiang Chen.

**Writing – original draft:** Hsiao-Ching Yen.

**Writing – review & editing:** Hsiao-Ching Yen, Jiann-Shing Jeng, Chiung-Hua Cheng, Guan-Shuo Pan.
